# Fecal Distribution Changes Using Colorectal Ultrasonography in Older People with Physical and Cognitive Impairment Living in Long-Term Care Facilities: A Longitudinal Observational Study

**DOI:** 10.3390/healthcare6020055

**Published:** 2018-05-25

**Authors:** Shiho Tanaka, Koichi Yabunaka, Masaru Matsumoto, Nao Tamai, Hiroshi Noguchi, Mikako Yoshida, Gojiro Nakagami, Junko Sugama, Hiromi Sanada

**Affiliations:** 1 Department of Gerontological Nursing/Wound Care Management, Graduate School of Medicine, The University of Tokyo, 7-3-1 Hongo, Bunkyo-ku, Tokyo 1130033, Japan; shihot-tky@umin.ac.jp (S.T.); gojiron-tky@umin.ac.jp (G.N.); 2 Department of Imaging Nursing Science, Graduate School of Medicine, The University of Tokyo, 7-3-1 Hongo, Bunkyo-ku, Tokyo 1130033, Japan; kyabunaka-tky@umin.ac.jp (K.Y.); matsumotom-tky@umin.ac.jp (M.M.); mokka-tky@umin.ac.jp (M.Y.); 3 Global Nursing Research Center, Graduate School of Medicine, The University of Tokyo, 7-3-1 Hongo, Bunkyo-ku, Tokyo 1130033, Japan; 4 Department of Skincare Science, Graduate School of Medicine, The University of Tokyo, 7-3-1 Hongo, Bunkyo-ku, Tokyo 1130033, Japan; ntamai-tky@umin.ac.jp; 5 Department of Life Support Technology (Molten), Graduate School of Medicine, The University of Tokyo, 7-3-1 Hongo, Bunkyo-ku, Tokyo 1130033, Japan; hnogu-tky@umin.ac.jp; 6 Institute for Frontier Science Initiative, Kanazawa University, 5-11-80 Kodatsuno, Kanazawa, Ishikawa 9200942, Japan; junkosgm@mhs.mp.kanazawa-u.ac.jp

**Keywords:** constipation, elderly, ultrasonography, nursing, bowel-related nursing care, long-term care, cognitive dysfunction

## Abstract

Nurses encounter difficulties evaluating constipation in elderly people with physical and cognitive impairment. Transabdominal ultrasonography (US) has been used to evaluate fecal impaction or fecal quality. However, it is unclear whether colorectal US can evaluate constipation symptoms in older people. Using colorectal US, we continuously observed the elderly and clarified the relationship between patterns of fecal distribution changes and constipation symptoms in older people with physical and cognitive impairment at long-term care facilities. This study included patients aged ≥65 years with oral intake. US was performed once a day until the next defecation, and fecal hardness was assessed. US images were extracted and categorized. Then, patterns of fecal distribution changes in the colorectum were classified. Multiple logistic regression analysis was performed to examine related factors associated with a constipation pattern. Among 101 patients, US images of 95 patients were analyzed. In 74.4% of the patients, US showed continuation of reflection with acoustic shadow in the rectum, which was significantly associated with defecation on the bed. Of the patients with a continuous crescent-shaped reflection pattern (R3), 92.9% had hard stool. R3 was found to be significantly associated with a Mini-Mental State Examination score of ≤10. In most of the patients, US detected a continuation of reflection with acoustic shadow in rectal patterns, indicating fecal retention in the rectum. Point-of-care US can be used by nurses to visualize rectal fecal retention as constipation patterns in the older people with physical and cognitive impairment at long-term care facilities.

## 1. Introduction

Constipation is defined as a state in which feces cannot be defecated in sufficient quantity and with comfort [1]. In general, constipation is diagnosed based on symptoms according to the Rome IV diagnostic criteria [2]. To define constipation, two or more of the following symptoms should be present: straining at stool, lumpy or hard stool, sensation of incomplete evacuation, sensation of anorectal obstruction or blockage, need for manual maneuvers, and fewer than three bowel movements per week. The pathophysiology of constipation includes impairment of colonic transit function (i.e., slow transit constipation) and defecation disorders, such as difficult defecation, incomplete evacuation, and rectal hyposensitivity [3,4].

The prevalence of constipation in the elderly population is as high as 30% compared with that in younger populations [5,6,7]. Japan has become a super-aging society with increasing number of elderly people with cerebrovascular diseases and dementia. It could be considered that the number of elderly people who could not complain of subjective symptoms or who have difficulty with communication are increasing.

Generally, in hospitals, nurses assess constipation in older people who have difficulty communicating their constipation symptoms accurately, based on the hardness of stool and bowel movement frequency, and choose bowel-related nursing care, such as laxative or suppository administration or digital disimpaction [8]. However, in home-care settings, constipation may be difficult to assess in older people by observing feces because visiting nurses cannot always observe the feces directly. Several problems such as diarrhea can be caused by administration of laxatives to patients who are not constipated [9], and administration of suppositories to patients without rectal fecal retention is inappropriate [1]. Furthermore, digital disimpaction should not be performed in patients without fecal impaction [10] because of its invasiveness and risk of rectal mucosa damage [11]. Therefore, to accurately assess constipation, a new objective assessment method for nurses to observe feces and evaluate constipation in home-care settings is needed.

Some objective assessment methods to observe feces in the body already exist, such as colon transit time examination (CTT), computed tomography (CT), and magnetic resonance imaging (MRI) [3,7]. Although CTT can evaluate the movement of feces, it cannot evaluate fecal properties and volume in real time. CT and MRI can evaluate fecal properties and volume; however, these methods are invasive because of radiation exposure or longer examination time. Moreover, the assessment methods as described above cannot be used for assessment by nurses in home-care settings.

In this study, transabdominal ultrasonography (US) was evaluated as an assessment method that nurses can use in home-care settings, as a US is easily portable and enables noninvasive observation of feces in real time [12,13]. Rectal US has already been used to evaluate the presence or absence of feces and fecal volume in children [14,15]. Moreover, colonic US can be used for qualitative assessment of feces in adults [13]. However, to our knowledge, previous studies have not clarified the relationship between the patterns of fecal distribution changes and constipation symptoms in the colorectum in elderly people. It is necessary to clarify the actual condition in facilities where feces can be directly observed.

The aims of this study were to continuously observe fecal distribution changes using colorectal US and to clarify the relationship between patterns of fecal distribution changes and constipation symptoms in older people with physical and cognitive impairment at long-term care facilities.

## 2. Materials and Methods

### 2.1. Study Design and Setting

This longitudinal observational, noninterventional study was conducted at two Japanese long-term care facilities from April to September 2017.

### 2.2. Patients

Patients aged ≥65 years with oral intake and ≥1 week of expected hospitalization were included in this study. Patients with a history of colorectal diseases and diarrhea-causing diseases (e.g., enteritis) and in whom US was difficult to perform were excluded.

### 2.3. Procedure

All inpatients were recruited at the facilities. The purpose and the protocol of the study were explained to the patients and their immediate families or extended relatives to obtain informed consent.

US was performed once a day in bed after the initial defecation until the next defecation. A US image of each part of the colon (the ascending, transverse, descending, and sigmoid colon) and the rectum was obtained. The patients and nurses were asked to report when defecating. A researcher checked the feces using the Bristol stool scale [16] immediately after defecation.

### 2.4. Measurements

#### 2.4.1. US Observation

A laptop-type ultrasonic device (Fujifilm FC-1; Fujifilm, Tokyo, Japan) with a curved array (2–5 MHz) transducer was used. The gain and image depth were adjusted as necessary.

The colorectum of all patients was scanned using a systematic scanning method [17,18] by a researcher who was a nurse trained in US. The US procedure was performed as follows: the colorectum was visualized using landmarks (iliac crest for the ascending and descending colon, abdominal midline for the transverse colon, up to the portion just beyond the left iliopsoas muscle for the sigmoid colon, and the suprapubic rim for the rectum). The colonic US image was captured by longitudinal scan [13], and the rectal US image was captured by transverse scan [14,15].

#### 2.4.2. Evaluation of Constipation Symptoms

Constipation symptoms were defined as less than one bowel movement every three days or hard stool (type 1 or 2 according to the Bristol stool scale) [2]. In this study, we observed patient bowel movements and directly evaluated stool properties.

#### 2.4.3. Variables Associated with Bowel Movement

Patient characteristics (including age, sex, body mass index, daily life independence level, diet intake, and drug use) and bowel-related nursing care were collected from medical records. Bowel-related nursing care included administration of oral laxatives (types and use), suppositories (use or no use), digital disimpaction (use or no use), and excretion place (on the bed, commode, or toilet). Then, we calculated the average dietary intake per meal during the observation period. We also classified the drug causing the constipation, including anticholinergics, antipsychotics, and opioids [1].

In addition, we individually evaluated the Barthel index and Mini-Mental State Examination (MMSE). A MMSE score of ≤10 was considered to indicate severe cognitive impairment [19].

### 2.5. Data Analysis

#### 2.5.1. Qualitative Analysis for US Images and Fecal Distribution Changes

Each US image finding was described according to morphoqualitative analyses [20] in the colon and rectum due to differences in each function. Descriptions were divided into codes. We grouped multiple codes and then categorized them. Second, we focused on the relationship among the categories and formed features of the US images. Third, the features were applied to colorectal parts in each patient. Finally, we grouped the multiple fecal distribution changes and generated patterns.

To ensure reliability, co-researchers with experience in qualitative analysis or sonographic analysis supervised the classification.

#### 2.5.2. Validation of Features

A text mining technique was applied for descriptions to validate the generated features. First, descriptions in a subcategory were parsed. Then, term frequency–inverse document frequency values were calculated per description as a feature vector. Finally, the scripts were classified by the k-means method, a type of clustering method [21]. The number of matched values between human and computer was used for validation. The text mining was performed using originally-developed software written in python 3.6 with open source software Juman ++1.02 and scikit-learn 0.19. 

#### 2.5.3. Relationship between Patterns of Fecal Distribution Changes and Constipation Symptoms

The relationship between patterns of fecal distribution changes and constipation symptoms was analyzed using Fisher’s exact test. Next, related factors between patterns of fecal distribution changes were compared using the Wilcoxon rank sum or Fisher’s exact test. A multiple logistic regression analysis was used to estimate odds ratio (OR) and 95% confidence interval of related factors contributing to patterns of fecal distribution changes. Variables with *p* values of < 0.05 in the univariate analysis and age, sex, and BMI were selected as factors to be entered into the multiple logistic regression analysis. Statistical analyses were performed using the JMP Pro 13.0 (SAS Institute Japan Ltd., Tokyo, Japan), and the level of statistical significance was set at a *p* value of < 0.05.

### 2.6. Ethical Consideration

This study was approved by the Research Ethics Committee of the Graduate School of Medicine, The University of Tokyo (#11521), and the ethics committees of the facilities. All participants and their families were advised that they were free to withdraw their consent at any time, and a researcher frequently observed adverse events during the study. The researcher immediately reported any abnormality to a clinical nurse and doctors.

## 3. Results

### 3.1. Flow and Characteristics of Patients

**Figure 1:**
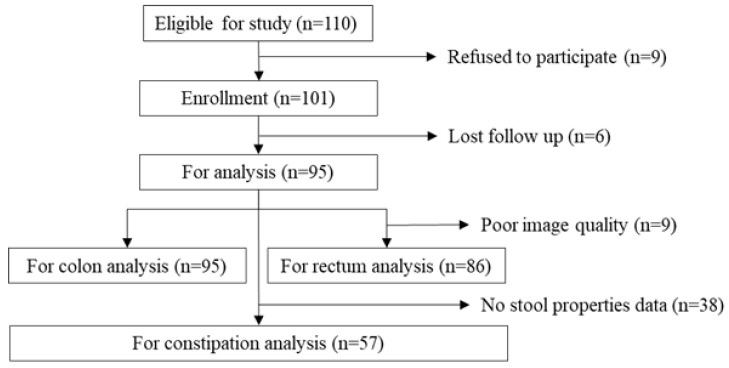
Flow chart of participant recruitment.

Of 110 eligible patients, 101 participated in this study. Six patients were excluded from analysis because of loss to follow-up. Of the remaining 95 patients, nine were excluded from rectal image analysis due to poor image quality, whereas 38 were excluded from the constipation analysis due to inability to evaluate fecal properties (Figure 1). Patient characteristics are shown in Table 1. Median patient age was 86 years (interquartile ratio (IQR), 80–91), and 55 patients (57.9%) were female. Median MMSE score was 11 (IQR, 0–20) and 84 (88.4%) patients were bedridden. Less bowel movement frequency was observed in 30 (52.6%) patients, and hard stool was observed in 19 (33.3%).

### 3.2. US Findings and Fecal Distribution Changes

**Figure 2:**
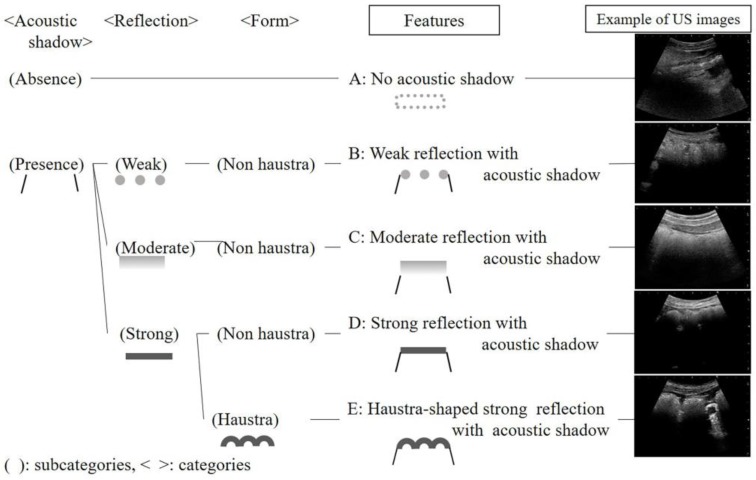
Features of ultrasonographic images in the colon.

First, we obtained a total of 1200 US images of each colon part (the ascending, transverse, descending, and sigmoid colon) from 95 patients. Three categories of acoustic shadow, reflection and form were formed from seven subcategories. From the combinations of the categories, five features were generated: (A) no acoustic shadow, (B) weak reflection with acoustic shadow, (C) moderate reflection with acoustic shadow, (D) strong reflection with acoustic shadow, and (E) haustra-shaped strong reflection with acoustic shadow (Figure 2).

**Figure 3:**
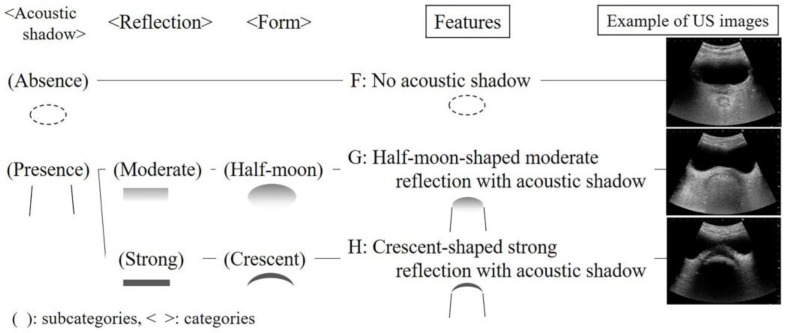
Features of ultrasonographic images in the rectum.

Next, we obtained a total of 284 US images of the rectum from 86 patients. Three categories of acoustic shadows, reflection, and form were formed from six subcategories. From the combinations of the categories, three features were generated: (F) no acoustic shadow, (G) half-moon-shaped moderate reflection with acoustic shadow, and (H) crescent-shaped strong reflection with acoustic shadow (Figure 3).

**Figure 4:**
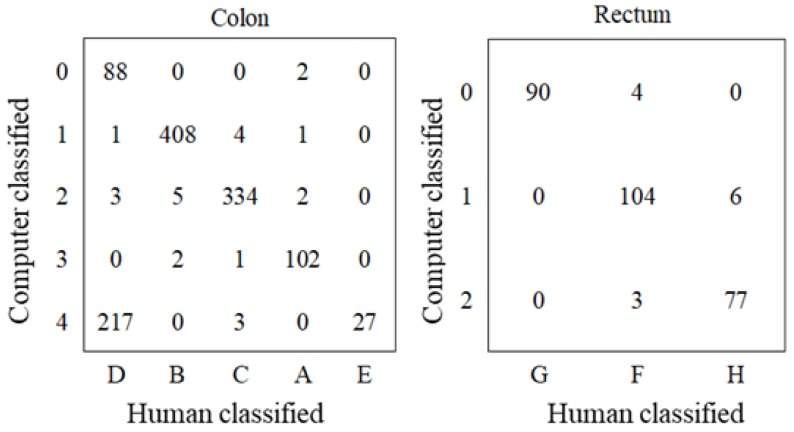
Matches of computer and human classifications.

The result of text mining analysis is shown in Figure 4. The numbers that matched with a human category were A, 102/107 (95.3%); B, 408/415 (98.3%); C, 334/342 (97.7%); D, 88/309 (28.5%); and E, 27/27 (100%). As for the rectum, the numbers were F, 104/111 (93.7%); G, 90/90 (100%); and H, 77/83 (92.8%).

### 3.3. Patterns of Fecal Distribution Changes and Constipation Symptoms

**Figure 5:**
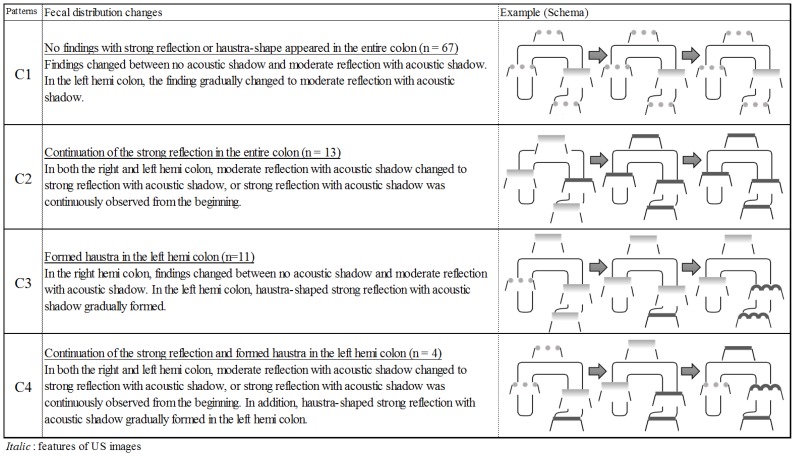
Patterns of fecal distribution changes in the colon (*n* = 95).

**Figure 6:**
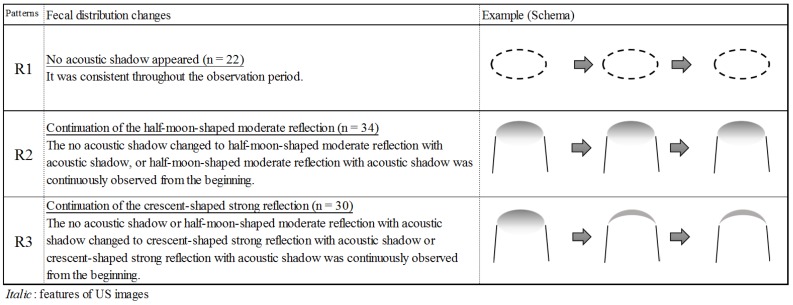
Patterns of fecal distribution changes in the rectum (*n* = 86).

Using image features, we classified four patterns of fecal distribution changes in the colon (Figure 5) and three patterns of fecal distribution changes in the rectum (Figure 6). A total of 64 patients (74.4%) had US findings with acoustic shadow (R2, 3) in the rectum. Next, the relationship between patterns of fecal distribution changes and constipation symptoms was analyzed. The patterns of fecal distribution changes in the colon were significantly associated with less bowel movement frequency. All patients with a C3 or C4 colon pattern showed less bowel movement frequency (Table 2). In addition, the patterns of fecal distribution changes in the rectum were significantly associated with less bowel movement frequency and hard stool. Of the patients with an R3 pattern, 13 (92.9%) had hard stool (Table 3). On the other hand, although the proportion of patients with constipation symptoms was a few, the R2 rectal pattern might indicate rectal fecal retention [15]. Based on these results, we considered that the patterns of C3 or C4 in the colon and R2 or R3 in the rectum indicated constipation patterns.

### 3.4. Related Factors of Constipation Patterns in the Rectum

The combinations of constipation patterns in the colon and rectum are shown in Table 4. Only three patients (5.3%) had constipation patterns in the colon (C3 or C4) and non-constipation patterns in the rectum (R1). Consequently, we focused on constipation patterns in the rectum.

To analyze the risk factors of patterns of rectal fecal retention, the rectal patterns were divided into R2 or R3 patterns and others (R1). R2 or R3 rectal patterns indicated significant differences in stool properties, MMSE scores, Barthel index, and excretion place as compared with R1 pattern (Table 5). A multiple logistic regression analysis was used to relate factors of patterns of rectal fecal retention. The MMSE score, Barthel index, and excretion place were multi-collinear with each other. Severe cognitive impairment (MMSE ≤ 10) and excretion on the bed were significantly associated with patterns of rectal fecal retention (adjusted OR = 4.7, *p* = 0.02; adjusted OR = 4.1, *p* = 0.02, respectively; Table 6).

To analyze the risk factors of constipation pattern related to hard stool, rectal patterns were divided into R3 constipation pattern and others (R1, R2). R3 constipation pattern related to hard stool showed significant differences in bowel movement frequency, hard stool, MMSE score, and Barthel index compared with other patterns (Table 7). A multiple logistic regression analysis was used to relate factors of R3 constipation pattern related to hard stool. The Barthel index was multi-collinear with MMSE. Severe cognitive impairment (MMSE ≤ 10) was significantly associated with the R3 constipation pattern related to hard stool (adjusted OR = 11.1, *p* = 0.01; Table 8).

## 4. Discussion

This study aimed to firstly investigate the usefulness of US for continuous observation of the patterns of fecal distribution changes in older people with physical and cognitive impairment, because US has been generally only used for the diagnosis of colorectal disease.

In healthy adults, rectal fecal retention was observed only when they had a desire to defecate [22]. However, in this study, rectal patterns of R2 or R3 were recognized as fecal retention [15] in approximately 70% of all patients. Further, the rectal fecal retention pattern was significantly associated with severe cognitive impairment and defecation on the bed. Thus, older people with physical and cognitive impairment tend to have fecal retention in the rectum (anorectal dysfunction). This indicates that abnormal patterns of fecal distribution changes existed. The older people may feel uncomfortable due to continuous fecal retention in the rectum. Elderly people with physical and cognitive impairment tend to have a higher rectal perception threshold than younger people [23], decreased rectal sensitivity and increased rectal compliance can contribute to fecal retention by decreasing the frequency and intensity of the desire to defecate [4]. Moreover, having a correct posture is crucial for appropriate defecation [24].

In this study, a new US finding was the R2 pattern, which indicated soft stool in approximately 80% of patients. The R3 pattern indicated hard stool in approximately 90% of patients and was significantly associated with severe cognitive impairment. The hardness of stool correlates with the length of bowel movement frequency [16]. As cognitive impaired patients have difficulty in perceiving the rectal fecal retention, or reporting a desire to defecate, feces should be stagnated, and the frequency in bowel movements should be longer. Therefore, rectal fecal properties should be assessed in patients with severe cognitive impairment using rectal US as hard stool is likely to be retained in the rectum. This can be assessed through the crescent-shaped strong reflection with an acoustic shadow.

A clustering method in the text mining was used for internal validation of qualitative analysis. This method is commonly used in information science areas for automatic categorization. The method is capable of validating generated features as another evaluator. The human-detected features were well matched with the computer-detected features except in one category. Features D (strong reflection with acoustic shadow) and E (haustra-shaped strong reflection with acoustic shadow) were similar because descriptions of both features included almost the same words, and the difference was in only a few words related to haustra. Thus, the computer could not distinguish well between the two features, which led to failure of detecting feature D. The consistency between human and computer feature detection indicated that the detected features might be valid.

**Figure 7:**
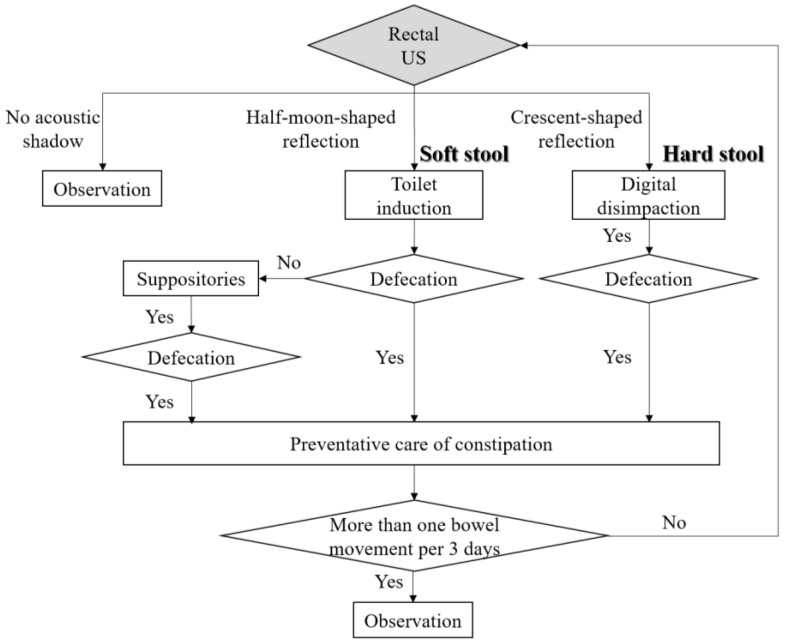
Bowel-related nursing care algorithm using US for older people with physical and cognitive impairment.

As a result of this study, nurses should assess constipation by US in older people with severe cognitive impairment and select appropriate bowel-related nursing care. Based on the results of this study, we propose a bowel-related nursing care algorithm using US for older people with physical and cognitive impairment (Figure 7). First, US should be performed to detect crescent-shaped strong reflection findings in the rectum because fecal impaction must be relieved [24]. Next, if rectal US detects a half-moon-shaped moderate reflection, nurses should select appropriate bowel-related nursing care, such as toilet induction, to promote defecation. After defecation, nurses should perform preventative care of constipation. Introducing this algorithm using US, it may be possible to assess fecal retention of hard stool in the rectum in older people without digital disimpaction. It contributes to patient’s comfort, because of noninvasive assessment in real time. Further studies are warranted to determine whether this algorithm can be particularly applied to patients with physical and cognitive impairment in home-care settings. Furthermore, we believe that US can solve nurses’ dilemma in bowel-related nursing care to patients who cannot communicate their desire to defecate.

Generally, US depends on operator skill and technique. However, this study has additional finding that nurses who were not familiar to US operation could detect easily the rectal fecal retention using US, according to this study process. The screening technique is as follows: first, US could be performed by applying the convex probe onto the suprapubic rim and further inclining the probe approximately 15° toward the head side to identify the rectum, as point-of-care US [25]. Moreover, US is considered as a relatively low-cost equipment, which requires lesser electricity and cost-effective echo jelly. Therefore, US can be used as a feasible assessment tool by nurses.

There are some limitations to this study. First, patients who have not defecated for 3 days had undergone routine care with administration of a suppository at the research hospitals. Thus, the results of this study may not be applicable at acute care hospitals. Second, the sample size of patients who could be observed of fecal properties was small. However, in this study, US was useful to objectively evaluate constipation in older people with physical and cognitive impairment.

## 5. Conclusions

US was continuously performed to determine patterns of fecal distribution changes in older people with physical and cognitive impairment. Point-of-care US can be used by nurses to visualize rectal fecal retention as constipation patterns. In 74.4% of all patients, US detected a continuation of reflection with acoustic shadow in rectal patterns, indicating fecal retention in the rectum. The patterns of rectal fecal retention were significantly associated with severe cognitive impairment and excretion on the bed. The pattern of continuation of crescent-shaped strong reflection with acoustic shadow was a characteristic pattern indicating hard stool and was significantly associated with severe cognitive impairment.

## Figures and Tables

**Table 1:** Patient characteristics (*n* = 95).

Variables	Categories	*n* (%)/Median (IQR ^1^)
Age		86.0 (80–91)
Sex	Male	40 (42.1)
	Female	55 (57.9)
BMI		19.0 (17.6–21.9)
Length of stay (days)		189.0 (51–729)
Main disease (ICD-10)	Disease of circulatory system	28 (29.5)
	Diseases of the respiratory system	20 (21.1)
	Mental and behavioral disorders	12 (12.6)
	Injury	9 (9.5)
	Metabolic diseases	7 (7.4)
	Neoplasms	3 (3.2)
	Diseases of the musculoskeletal system	3 (3.2)
	Diseases of the genitourinary system	3 (3.2)
	Diseases of the nervous system	1(1.1)
	Others	9(9.5)
MMSE ^2^		11 (0–20)
Barthel index		10 (5–40)
The daily life independence level ^3^	Grade J or A	11 (11.6)
	Grade B or C	84 (88.4)
Dietary intake ratio (total diet) (%)		94.0 (79–100)
Medication	Anticholinergics drugs	5 (5.3)
	Psychotropic drugs	19 (20.0)
	Opipod	2 (2.1)
	Antiparkinsonian drugs	5 (5.3)
	Cardiovascular drugs	56 (59.0)
	Anticoagulants/Antiplatelets	20 (21.1)
	Sleeping drugs	19 (20.0)
	Diuretics	31 (32.6)
	Probiotics	9 (9.5)
Bowel-related care	Suppository	26 (27.4)
	Digital disimpaction	21 (22.1)
	Osmotic laxative	33 (34.7)
	Stimulant laxative	16 (16.8)
	Abdominal massage	1 (1.1)
	Hot compress	0 (0.0)
Excretion place	Bed	59 (62.1)
	Commode	22 (23.2)
	Toilet	14 (14.7)
Bowel movement frequency ^4^	Less	30 (52.6)
Stool form ^5^	Hard	19 (33.3)

^1^ IQR: Interquartile range. ^2^ MMSE: Mini-Mental State Examination. ^3^ J: independent, A: need assistance for going outside, B: need assistance to daily life but sitting position is possible, C: Living on the bed and need assistance in daily life. ^4^ Less: less than one bowel movement per three days. ^5^ Hard: type 1 or 2 according to the Bristol stool scale.

**Table 2:** Relationship between the patterns of fecal distribution changes in the colon and constipation symptoms (*n* = 57).

US Patterns	*n*	Less BM Frequency ^1^		*p* Value	Hard Stool ^2^		*p* Value
C1	39	14	(35.9)	0.00 *	10	(25.6)	0.19 *
C2	8	6	(75.0)		5	(62.5)	
C3	7	7	(100.0)		3	(42.9)	
C4	3	3	(100.0)		1	(33.3)	

*n* (%). *p* values for Fisher’s exact test. *: *p* < 0.05. ^1^ Less BM frequency: less than one bowel movement per three days. ^2^ Hard stool: type 1 or 2 according to the Bristol stool scale.

**Table 3:** Relationship between the patterns of fecal distribution changes in the rectum and constipation symptoms (*n* = 57).

US Patterns	*n*	Less BM Frequency ^1^		*p* Value	Hard Stool ^2^		*p* Value
R1	21	8	(38.1)	0.00 *	3	(14.3)	0.00 *
R2	22	9	(40.9)		3	(13.6)	
R3	14	13	(92.9)		13	(92.9)	

*n* (%). *p* values for Fisher’s exact test. *: *p* < 0.05. ^1^ Less BM frequency: less than one bowel movement per three days. ^2^ Hard stool: type 1 or 2 according to the Bristol stool scale.

**Table 4:** Combinations of constipation patterns in the colon and rectum (*n* = 57).

Patterns of Fecal Distribution Changes			
Colon ^1^	Rectum ^2^	*n*	(%)
−	−	18	(31.6)
+	−	3	(5.3)
−	+	29	(50.9)
+	+	7	(12.3)

^1^ Colon+: haustra patterns (C3 or C4), Colon−: others (C1 or C2). ^2^ Rectum+: presence of acoustic shadow (R2 or R3), Rectum−: the other (R1).

**Table 5:** Relationship between the patterns of rectal fecal retention and other related factors (*n* = 57).

Variables	Categories	US Patterns		*p* Value
		R1 (*n* = 21)	R2, 3 (*n* = 36)	
Age		84 (67–98)	86 (66–99)	0.35
Sex	Male	13 (61.9)	15 (41.7)	0.18
	Female	8 (38.1)	21 (58.3)	
BMI		19.2 (13.5–30.7)	19.1 (11.5–31.5)	0.47
MMSE ^1^	>10	15 (71.4)	14 (38.9)	0.03 *
	≤10	6 (28.6)	22 (61.1)	
Barthel index		40 (0–85)	10 (0–80)	0.01 *
Daily life independence level ^2^	J, A	6 (28.6)	4 (11.1)	0.15
	B, C	15 (71.4)	32 (88.9)	
Excretion place	Toilet or commode	14 (66.7)	7 (19.4)	0.03 *
	Bed	7 (33.3)	23 (63.9)	
Diet intake ratio		9.2 (0.1–10)	9.7 (1.8–10)	0.30
Drug causing constipation ^3^	No use	15 (71.4)	25 (69.4)	1.00
	Use	6 (28.6)	11 (30.6)	
Laxative ^4^	No use	8 (38.1)	23 (63.9)	0.10
	Use	8 (38.1)	22 (61.1)	
Bowel movement frequency ^5^	Normal	13 (61.9)	14 (38.9)	0.11
	Less	17 (81.0)	13 (36.1)	
Stool form ^6^	Not hard	18 (85.7)	20 (55.6)	0.02 *
	Hard	3 (14.3)	16 (44.4)	

Median (range) or *n* (%). *p* values for Wilcoxon rank sum test or Fisher’s exact test. *: *p* < 0.05. ^1^ MMSE: Mini-Mental State Examination. ^2^ J: independent, A: need assistance for going outside, B: need assistance to daily life but sitting position is possible C: Living on the bed and need assistance in daily life. ^3^ Drug causing constipation: anticholinergics drug, psychotropic drug, and/or Opioid. ^4^ Laxative: osmotic laxative and/or stimulant laxative. ^5^ Less: less than one bowel movement per three days. ^6^ Hard stool: type 1 or 2 according to the Bristol stool scale.

**Table 6:** Multiple logistic regression analysis of the patterns of rectal fecal retention (*n* = 57).

Variables	Categories	OR	95% CI	*p* Value	Model 1 ^1^ AOR	95% CI	*p* Value	Model 2 ^2^ AOR	95% CI	*p* Value	Model 3 ^3^ AOR	95% CI	*p* Value
Age		1.0	(1.0–1.1)										
Sex	Male	1.0											
	Female	2.3	(0.8–6.8)										
BMI		1.0	(0.8–1.1)										
Barthel index		1.0	(1.0–1.0)										
MMSE	>10	1.0			1.0						1.0		
	≤10	3.9	(1.2–12.5)	0.02 *	4.7	(1.3–16.8)	0.02 *				3.2	(0.9–12.9)	0.07
Excretion place	Toilet or Commode	1.0						1.0			1.0		
	Bed	3.5	(1.1–11.0)	0.03 *				4.1	(1.2–13.7)	0.02 *	2.8	(0.8–10.3)	0.12

AOR = adjusted odds ratio, CI = confidence interval. *: *p* < 0.05. ^1^ Model 1: adjusted for age, sex. BMI and MMSE. ^2^ Model 2: adjusted for age, sex, BMI and excretion place. ^3^ Model 3: adjusted for age, sex, BMI, MMSE and excretion place.

**Table 7:** Relationship between constipation patterns related to hard stool and other related factors (*n* = 57).

Variables	Categories	US Patterns		*p* Value
		R1, 2 (*n* = 43)	R3 (*n* = 14)	
Age		84 (66–98)	87 (70–99)	0.08
Sex	Male	23 (53.5)	5 (35.7)	0.36
	Female	20 (46.5)	9 (64.3)	
BMI		19.2 (12.0–31.5)	19.1 (11.5–26.7)	0.53
MMSE ^1^	>10	27 (62.8)	2 (14.3)	0.00 *
	≤10	16 (37.2)	12 (85.7)	
Barthel index		20 (5–45)	5 (0–26)	0.04 *
Daily life independence level ^2^	J, A	9 (20.9)	1 (7.1)	0.42
	B, C	34 (79.1)	13 (92.9)	
Excretion place	Toilet or commode	22 (51.2)	5 (35.7)	0.37
	Bed	21 (48.8)	9 (64.3)	
Diet intake ratio		9.6 (0.1–10)	9.8 (7.7–10)	0.37
Drug causing constipation ^3^	No use	31 (72.1)	9 (64.3)	0.74
	Use	12 (27.9)	5 (35.7)	
Laxative ^4^	No use	22 (51.2)	9 (64.3)	0.54
	Use	21 (48.8)	5 (35.7)	
Bowel movement frequency ^5^	Normal	26 (60.5)	1 (7.1)	0.00 *
	Less	17 (39.5)	13 (92.9)	
Stool form ^6^	Not hard	37 (86.0)	1 (7.1)	0.00 *
	Hard	6 (14.0)	13 (92.9)	

Median (range) or *n* (%). *p* values for Wilcoxon rank sum test or Fisher’s exact test. *: *p* < 0.05. ^1^ MMSE: Mini-Mental State Examination. ^2^ J: independent, A: need assistance for going outside, B: need assistance to daily life but sitting position is possible C: Living on the bed and need assistance in daily life. ^3^ Drug causing constipation: anticholinergics drug, psychotropic drug, and/or Opioid. ^4^ Laxative: osmotic laxative and/or stimulant laxative. ^5^ Less: less than one bowel movement per three days. ^6^ Hard stool: type 1 or 2 according to the Bristol stool scale.

**Table 8:** Multiple logistic regression analysis of the constipation pattern related to hard stool (*n* = 57).

Variables	Categories	OR	95% CI	*p* Value	Model 1 ^1^ AOR	95% CI	*p* Value	Model 2 ^2^ AOR	95% CI	*p* Value
Age		1.1	(0.9–1.2)							
Sex	Male	1.0								
	Female	2.1	(0.6–7.2)							
BMI		0.9	(0.8–1.1)							
MMSE	>10	1.0			1.0			1.0		
	≤10	10.1	(2.0–51.1)	0.01 *	11.1	(2.1–60.2)	0.01 *	9.7	(1.5–65.2)	0.02 *
Barthel Index		1.0	(0.9–1.0)	0.08				1.0	(1.0–1.0)	0.77

AOR = adjusted odds ratio, CI = confidence interval. *: *p* < 0.05. ^1^ Model 1: adjusted for age, sex. BMI and MMSE. ^2^ Model 2: adjusted for age, sex, BMI, MMSE and Barthel index.
